# Posterior uterine rupture secondary to use of herbs leading to peritonitis and maternal death in a primigravida following vaginal delivery of a live baby in western Uganda: a case report

**DOI:** 10.11604/pamj.2016.23.81.9044

**Published:** 2016-03-11

**Authors:** Jonathan Peter Nelson

**Affiliations:** 1 Fort Portal Regional Referral Hospital, Fort Portal, Uganda

**Keywords:** Maternal death, uterine rupture, global health, maternal health, herbal medicines

## Abstract

Uterine rupture is a potentially avoidable complication resulting in poor perinatal and maternal outcomes. This case had a number of unusual features including delivery of a healthy live baby; spontaneous posterior uterine rupture in a primigravida (and unscarred uterus); and delayed presentation with signs of peritonitis and sepsis rather than haemorrhage. A 19-year old primigravida had a vaginal delivery of a live infant at term, reporting having taken herbs to induce labour. She deteriorated and was transferred to our unit where she was found to have reduced consciousness, a distended abdomen and signs of sepsis. At laparotomy there was blood-stained ascites, signs of peritonitis and a posterior lower segment uterine rupture. A sub-total hysterectomy was performed but the patient's condition worsened resulting in maternal death 5 days post-operatively. This case highlights a number of differences in the presentation, management and outcomes of uterine rupture in resource-poor compared to resource-rich countries.

## Introduction

Uterine rupture is the complete disruption of all layers of the uterus, including the serosa. In resource-rich countries the most significant risk factor is a previous caesarean section, where the risk of rupture is put at 22-74/10,000 with one previous scar [1], as compared to 0.5-2.0/10,000 in unscarred uteri [2]. However, in resource-poor countries the aetiology is also related to prolonged and obstructed labour, and rupture occurs more commonly in unscarred uteri. This results in a much higher incidence, such as 1 in 131 as reported by one study in Uganda [3]. This difference in aetiology and incidence is accompanied by a greater number of reported risk factors in resource-poor countries. These include previous scar, but also grand multi parity, use of herbs, referral from another health centre and distance travelled to hospital [3]. Rupture of a primigravida, especially one who has not undergone previous uterine surgery is extremely rare. Ekpo reported the proportion to be 5% of ruptures in resource-poor countries [4]. In this study, rupture in a primigravida represented 4 cases, 3 of which were traumatic due to instrumental delivery or destructive procedures. Only one was spontaneous. Use of herbs is a recognised risk factor in certain regions of Africa. In a study from another referral hospital in Western Uganda [3] the odds ratio of having a rupture following use of herbs was 15.2, being reported in 35% of cases. Traditional herb use for medicinal purposes is widespread in Western Ugandan, and one survey suggested that up to 80% of childbirths occur at home using these herbs for induction [5]. The obvious danger lies in the variability of the effects and dosages administered.

Presentation can also differ when comparing resource-rich and resource-poor countries. Due to increased monitoring the first sign of uterine rupture in resource-rich countries is often fetal heart rate abnormalities such as decelerations and bradycardia [6], followed by abdominal pain from the rupture, vaginal bleeding (although this may be modest) and intra-abdominal haemorrhage, and finally shock. Fetal parts may be felt more prominently due to extrusion of the fetus into the abdominal cavity, but the diagnosis is confirmed at laparotomy. In resource-poor countries due to delay in attending and lack of monitoring it is more likely to present later in this sequence, with maternal shock and fetal death. For example, in Uganda maternal death from rupture may be 10%, and perinatal death 80%, compared to 0.3% [7] and 5% [8] respectively in resource-rich countries. The aetiology of these poor maternal outcomes is usually related to haemorrhage, and often bleeding is concealed in the abdominal cavity. Management of the suspected uterine rupture usually involves rapid transfer to theatre for exploratory laparotomy, although resuscitation may be needed first. At laparotomy the surgeon usually has a choice between hysterectomy and repair of the rupture, and this will depend upon the clinical circumstances and condition of the patient. Hysterectomy is used in 65% of cases [3] in resource-poor countries, as compared to uterine repair in the majority of cases in resource-rich countries. This probably reflects some of the differences already discussed, such as the proportion of ruptures in patients with a previous scar and the timing of presentation/recognition of the rupture. Site of rupture is most commonly along the site of a previous scar if it is present e.g. anteriorly in the lower segment. However, in unscarred uteri rupture can be lateral, fundal or posterior. For example, a study from Nigeria reported that 18% of ruptures were posterior [4]. Some definitions of uterine rupture include the finding of the fetus in the abdomen. However, uterine rupture can occur following a vaginal delivery, with one study showing 3 of 19 uterine ruptures identified at or after delivery [9]. This case has a number of unusual features that appear to make it unique in the literature. These features are the vaginal delivery of a live baby with no adverse outcomes; spontaneous rupture in a primigravida (and unscarred uterus); posterior uterine rupture; and delayed presentation with signs of peritonitis and sepsis rather than haemorrhage.

## Patient and observation

The patient was a 19-year old female primigravida who presented with labour-like pains to her local health centre. She was thought to be at term based on her last menstrual period, and had attended one antenatal appointment (of the usual four that are offered). She reported no previous medical or surgical history and screening test showed she was HIV negative. She is reported to have told staff that she took "herbs" to start (or induce) her labour.

Following a vaginal delivery of a live baby of normal weight she had a post-partum haemorrhage and complained of abdominal pain, but initially this was managed in the local health centre. However, after 24 hours she was seen to be deteriorating with confusion and decreased conscious level so was transferred to our unit, the referral hospital for the region. Here an intern doctor assessed her and made a provisional diagnosis of sepsis of unknown origin and started the patient on broad-spectrum intravenous (IV) antibiotics and fluids. At this stage no observations were recorded and no formal scale was used to assess her conscious level, although it was commented on that she "unresponsive". Again, no documentation was made as to whether she was given the treatment prescribed. The following morning she was reviewed by the author, now more than 48 hours since delivery. The patient's observations were pulse rate 110 beats per minute, respiratory rate 50 breaths per minute, blood pressure 110/75 mmHg and oxygen saturations were 82% on air. No temperature was recorded as no thermometer was available in the unit. She appeared clinically dehydrated, with concentrated urine, and her GCS was 7. On examination the chest had bilateral basal crepitations, heart sounds were normal and there was a distended abdomen. On vaginal examination there was minimal lochia and it was non-offensive. She was given oxygen therapy at the highest concentration available (4L/min via nasal prongs) and saturations improved to 92%. The patient was given IV antibiotics, 2 litres of IV normal saline and a decision was made to perform an abdominal ultrasound scan. This was done quickly and the only positive findings were of significant free fluid/haemoperitoneum. At this point the diagnosis was sepsis with possible uterine rupture. A decision was made for exploratory laparotomy.

A sub-umbilical midline incision was performed and at laparotomy there were findings of 2-3L of blood stained ascites, sloughy exudate all over the intra-abdominal organs, and a posterior uterine rupture in a transverse direction approximately 7cm in width. The site of the rupture was very low 1-2cm above the internal os of the cervix, although it had not extended into the cervix, vagina or laterally. In view of the viability of the uterine tissue and site of rupture a sub-total hysterectomy was performed and the abdomen washed-out and closed. In the following 24 hours the patient's condition continued to deteriorate and although her urine output improved with the IV fluids, her conscious level did not improve, and in-fact deteriorated to a GCS of 5 and then 3. Treatment was mainly palliative from then on and the patient died a few days later.

## Discussion

It is important to comment on the level of care available in our setting. Fort Portal Regional Referral Hospital is a government hospital taking referrals from a wide area. There are significant limits on human resources, the level of care available and the equipment in the hospital. We were unable to perform any blood or microbiology tests on this patient at any stage of her care due to laboratory problems. There is no recognised higher level of care, for example ventilation or intensive care facilities. Diagnostic tests are also limited, with ultrasound and x-ray the only imaging available. The only oxygen available is via oxygen concentrators and nasal cannula. Having said this, there was a delay in the patient being transferred to our facility, in initiating and maintaining treatment, and in senior medical review. Despite this the rapid deterioration of the patient was unexpected give her age and medical history. The most likely explanation (again no post-mortem was available) would be sepsis secondary to uterine perforation and the unrecognised perforation acting as a site for genital tract pathogens to enter the peritoneal cavity. Usually haemorrhage is the most significant factor in maternal morbidity and mortality following uterine perforation, although sepsis may be more likely in resource-poor countries if there is a delay in seeking care.

One of the key factors in this case was the delay and difficulty in making the diagnosis. Uterine rupture was not initially suspected. This was because the patient did not have any of the "usual" risk factors such as previous uterine scar or grand multi parity. Also, she did not have significant clinical signs such as vaginal bleeding or signs of intra-abdominal haemorrhage. Her clinical deterioration, whilst quick over 48 hours, was not as rapid as often seen with uterine rupture.

Another reason for delay was the normal delivery of a live birth with no apparent compromise. This is unusual and, given the lack of other commonly recognised risk factors, rupture would appear unlikely. The lack of literature on rupture following vaginal delivery makes it difficult to comment with any authority. However, it does seem plausible that the site of rupture could have contributed to the fetus not being expelled into the uterine cavity. It appears logically that with a low posterior rupture there is limited space for the fetus to leave the uterus. Also, if the fetal head is low or close to delivery at rupture it is feasible that with the upper segment intact uterine contractility could be maintained enough to allow a vaginal delivery. In reviewing the literature on posterior rupture it is difficult to draw any conclusions, as it is limited to case reports.

## Conclusion

This case report highlights a number of differences in the presentation, management and outcomes of uterine rupture in resource-poor compared to resource-rich countries. The case had a number of unusual features that remind us of the need to consider uterine rupture even when there is an atypical presentation. It also highlights the importance of knowledge of a wide variety of risk factors, some of which may be specific to a local population.
